# Evolution of the Multielemental Content along the Red Wine Production Process from *Tempranillo* and *Grenache* Grape Varieties

**DOI:** 10.3390/molecules25132961

**Published:** 2020-06-27

**Authors:** Alexandra Bica, Raquel Sánchez, José-Luis Todolí

**Affiliations:** Department of Analytical Chemistry, Nutrition and Food Science, University of Alicante, P.O. Box 99, 03080 Alicante, Spain; alexandra.bica@gmail.com (A.B.); r.sanchez@ua.es (R.S.)

**Keywords:** red wine, winemaking, metals, rare earth elements, inductively coupled plasma, mass spectrometry, wine waste quality

## Abstract

In the present work, 38 elements were quantified in the different fractions generated by applying amateur winemaking methods. Inductively Coupled Plasma Mass Spectrometry was used as detection technique. Grapes were analyzed and separate metal profiles were also obtained for the skin and seeds. Additional vinification fractions included musts before and after the fermentation process. Meanwhile, solid fractions corresponded to the so-called hat, pressed pomace and the lees obtained after gravitational settling at the tank bottom. Wine was further analyzed. The obtained results revealed a different repartition depending on the particular element and winemaking solid and liquid fraction evaluated. The studies included vinification in presence and in absence of added yeast and grape geographical origin. Principal component analysis helped to discriminate among fractions and to determine the critical elements behaving differently. Finally, a mass balance allowed to unequivocally detect the migration of a given element to the winemaking fractions.

## 1. Introduction

The elemental composition of wine is an important issue affecting the quality, aging and geographical origin [1] of the obtained alcoholic beverage. In this context, there are two main sources of metals [2]: natural sources, including the root absorption from soil, and anthropogenic sources related with the harvest practices (i.e., use of fertilizers, phytosanitary treatments), winemaking process (e.g., type of reservoirs, conductions, use of additives) or environmental pollution [3]. For instance, metals such as Cd, Cu, Mn or Zn may be incorporated, as they are commonly present in pesticides, fungicides and fertilizers. A possible classification of elements present in wine is also based on primary (i.e., those that originate from the vineyard soil) and secondary that correspond to the elements being incorporated during the grape processing.

The characteristics of the wine depend, partially, on the metals present and their concentration. For instance, elements such as Al, Cu, Fe, Mn, Ni and Zn contribute to the final wine color, because they form organometallic complexes with anthocyanins and tannins [4]. High Cu and Fe levels may confer unpleasant flavor to the finally obtained wine and cause precipitates, especially at high pH values [5]. A similar phenomenon is observed at high Al concentrations [6]. Additionally, toxicological concerns exist for several elements, e.g., Pb, As or Cd [7]. In fact, the International Organization of Vine and Wine (OIV) has recommended maximum levels of these two elements, together with B, Br, Cu, Ag, Pb, Na and Zn [8]. Finally, it is indicated that wine also contributes to the intake of some essential elements such as Ca, Co, Cr, Cu, Fe, K, Mg, Mn, Mo, Ni or Zn.

Another important aspect is related to the potential uses of the residues issued from the winemaking process, e.g., pressed pomace, mark, lees [9]. From a compositional point of view, it can be indicated that 62% of the total mass of sub-products generated corresponds to the marc that contains water (roughly 60%) and skin (50% on a dry mass basis), seeds (47% on a dry mass basis) and stems [10]. These materials are of great environmental concern, because they are produced in a large amount during a short period of time [11]. Their possible applications include direct combustion to generate energy and biofuels [12], as well as compost production [13]. In fact, these materials can be considered an appropriate source of humic substances [14]. Additional interest has been paid to the use of marc as a source of antioxidants such as polyphenols or surfactants [15]. In addition, the high content of antioxidants in the seeds, together with the appropriate level of elements such as Ca, Mg, Na, K, Fe, Zn, Cu and Mn, make this product highly recommended as a supplement in animal feed [16].

In order to gain more control on the production process, thus increasing the quality and safety of the final product, it is very important to detect changes in the multielemental content of the different products. The level of metals should be known, and the evolution of their concentration must be followed. Note that metals may accumulate if the residues are incorporated to the soil.

Up to now, there have been a few studies dealing with the evolution of the elemental composition during the wine production process [17]. Furthermore, the list of evaluated elements is actually short, and the solid residues are not commonly analyzed, but only liquid fractions are of interest. In addition, there are a lack of studies on this subject dealing with Spanish red wines. The goal of the present work was thus to monitor the evolution of the content of metals, metalloids and rare earth elements during the winemaking process in a rural environment with an ecological (non-certified) cultivation method, in which bentonite, a recognized source of metals for wine, was not added as clarifying agent. A further aim of the present research was to fully characterize the residues generated from the point of view of elemental composition, which could help to assess the safety level of these products. Hypothetically, the contribution of the instruments used in the production process to the final elemental composition of the different fractions obtained could be detected through mass balance. This last point could help to detect critical points within the wine production process.

## 2. Results and Discussion

### 2.1. Method Validation

The first step was to test the reliability of the method for the elemental analysis selected in the present study. The efficiency of the microwave digestion step was verified by means of the measurement of the ICP-OES emission intensity at the C 193.030 nm line. By comparison with the corresponding signals for the standards or blanks, it emerged that the digests contained a significant fraction of dissolved organic matter (higher carbon emission intensities). This fact suggested that the digestion of the samples was incomplete and that ICP-MS interferences caused by the presence of carbon in the samples could occur. Therefore, a solution containing 50 μg kg^−1^ of ^45^Sc, ^72^Ge, ^103^Rh and ^185^Re was added online, in order to apply internal standardization, and to compensate for possible interferences caused by the sample matrix, as well as instrumental signal drift.

Limits of detection (LODs) were calculated according to the following equation:

(1) $$LOD=\frac{3\;s_{b}}{S}$$

where *s_b_* is the standard deviation of twenty consecutive measurements of a blank solution containing nitric acid and *S* is the slope of the calibration line.

Method limits of quantification (mLOQ) were also obtained by multiplying the limit of quantification, LOQ, found according to:

(2) $$LOQ=\frac{10\;s_{b}}{S}$$

by the dilution factor that, as detailed in Section 4, was 12.5. The obtained results are shown in Table S1 for all the elements quantified. The elements quantified in the present work were those whose concentrations were far above the method LOQs given in Table S1.

In order to verify the accuracy of the analytical procedure, samples were spiked with a multielemental standard at a 40 μg kg^−1^ level and the concentration was determined. It was verified that, within a 10% tolerance, the measured concentrations corresponded to the spiked ones. Further experiments were performed, in order to verify the accuracy of the method, and the concentrations obtained by means of ICP-OES were compared with those provided by the ICP-MS instrument. Again, good correlation between both techniques was obtained. In the present work, the elemental concentrations were expressed on a dry weight basis (i.e., *w*/*w*). Measured densities of the liquid fractions corresponded to 1070, 995 and 993 g L^−1^ for must, must after 8 days’ fermentation and wine, respectively. Taking these data into account, the elemental concentrations in liquid fractions, expressed in terms of *w*/*v*, were easily obtained.

### 2.2. Grape and Yeast Analysis

Once the analytical method was established, experiments were carried out with grape (a mixture of grapes belonging to the varieties *Tempranillo* and *Grenache* at 4:1 proportion) and yeast (*Saccharomyces cerevisiae*). As Figure S1a reveals, elements were more abundant in the grape skin than in the seeds, the overall grape concentration being intermediate between them. This trend has already been described in the literature, for elements such as Pb [18]. From these data, it was observed that some elements (B, Na, Mg, Al, P, K, Ca, Ti, Mn, Fe, Cu, Zn, Sr and Ba) were present at concentrations above 1000 μg kg^−1^ on a dry mass basis and, thus, could be considered micro- and macro-elements. Meanwhile, others were encountered at trace levels (V, Cr, Ni, As, Se, Rb, Mo, Cd, Sn, Ba, Hg and Pb), whose concentrations were on the order of several hundreds of micrograms per kilogram, or even lower.

A special comment should be made regarding the contents of rare earth elements (See Figure S1b). The relevance of these elements is related to the fact that they can give signatures of the wines according to their geographical origin [19]. In the present study, it was found that, these elements, whose concentrations were on the order of parts per trillion (i.e., nanograms per kilogram), also preferentially accumulated in the grape skin.

When the yeast was analyzed (Figure S1c), it was found that it was particularly rich in elements such as Na, Mg, P, K, Ca, Fe and Zn. Therefore, it was necessary to evaluate the contribution of the yeast used during the vinifying process to the final composition of both wine and the by-products of the winemaking process.

### 2.3. Evolution of the Content of Major Elements

**Figure 1:**
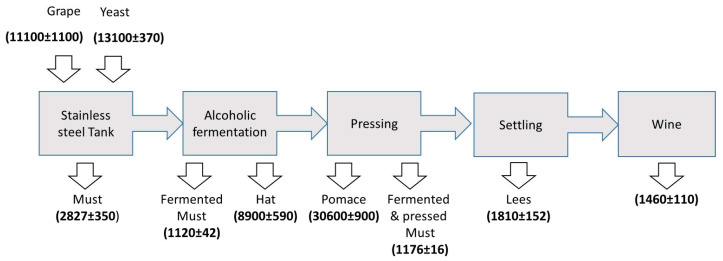
Evolution of potassium content (on a dry mass basis) along the winemaking process. The figures in bold correspond to the potassium concentration expressed as mg kg^−1^. Confidence intervals have been determined according to: $\pm \;\frac{t\;s}{\sqrt{n}}$ (*n* = 4; t for a 99% confidence level: 3.74).

As mentioned before, within the frame of the present work, ‘major elements’ corresponded to those whose concentrations in most of the fractions was above 1000 μg kg^−1^. The trend found for major elements is exemplified for potassium (Figure 1). Once the alcoholic fermentation was completed, this element was enriched in the solid fraction above the must (so called hat). According to the encountered data, the potassium concentration was around eight times higher in the solid residue, with respect to that in the fermented must. This was compatible with the fact that potassium was preferentially present in the bay skin, as was previously highlighted in Figure S1. The level of this element remained constant in the resulting must after the mixture was pressed, and the malolactic fermentation did not severely affect the potassium concentration (See Figure 1). In contrast, this element was present at an actually high concentration in the pressed pomace. Comparatively speaking, the concentration of potassium was around 25 times higher in the pressed solid fraction than in musts or wine. It is worth mentioning that yeast consume significant fractions of potassium during the fermentation process, thus lowering its concentration in the liquid fractions [20]. A slight increase in the concentration of this element was found in the decanted solids, as compared to the potassium concentration in the wine finally obtained. A comment should be made regarding the water content in the solid samples (e.g., 79 ± 3 and 82 ± 2% in the solids before and after pressing, respectively). Even if the concentrations of potassium were referred to wet mass, the comments made before were qualitatively valid.

Results corresponding to the remaining major elements are summarized in Table 1. The data in this table correspond to the winemaking process in which no yeasts were added. In this case, autochthonous yeasts existing on the bays skin were the responsible for the fermentation. The concentrations of elements such as boron, sodium or magnesium in the liquid fractions were fairly high. In contrast, aluminum, phosphorous, calcium, iron or copper were present in the liquid fractions at levels much lower and far below those encountered in the solid fractions of the winemaking process. The consumption of elements such as Ca, Mg, Fe, Cu and Zn by yeasts may explain their preferential accumulation in the solid fractions. Indeed, previous reports have demonstrated that a fraction of Zn present in must migrate, thus leaving wines with lower contents of this metal [21]. Table 1 also presents the confidence intervals. It should be pointed out that the solid fractions presented, in general terms, higher variability on a relative basis. Thus, the Relative Standard Deviation (RSD) of the concentrations were as high as 10–20% for solid fractions, whereas this parameter took values generally lower than 10% for liquid ones. A possible explanation could be the heterogeneity of the solid residues, as compared to the liquid fractions. 

An interesting trend emerged for elements that can act as promoters of some enzymes, or as catalysts of oxidation reactions, such as Fe, Cu and Zn, because they were more abundant in the wine than in the remaining liquid fractions. Among the possible reasons for this trend, the solubilization of a fraction of these elements or their presence as suspended microparticles could be argued. In the particular case of Cu, mainly originated from treatment with pesticides [22], it has been indicated that, under oxidative conditions, it may be scavenged by the solid fractions [23]. Consequently, the concentration of this element was high in the pressed pomace. 

For Mn, it has been indicated that its content does not change during the former fermentation steps [20], which is compatible with the fact that, in the present work, the concentration for this element was similar for fermented and pressed must and wine.

### 2.4. Evolution of the Content of Minor Elements

The results found for minor elements are included in Table 2. Nickel was the most abundant element in these series. In fact, as could be observed from Figure S1, this element was found at a rather high content in the grape (230 μg kg^−1^) and its skin (692 μg kg^−1^). As a result, Ni prevailed in the liquid fractions with concentrations close to 100 μg kg^−1^. It was clearly observed that nickel accumulated preferentially in both the hat and the solid pressed pomace. Chromium and vanadium were also present at remarkable concentrations, although their levels in liquid fractions were around five times lower than that for nickel. The remaining elements were detected at very low contents, although some interesting observations could be made. The former one was that the pressed pomace contained Co, As, Se and Hg in concentrations of tens of parts-per billion. Meanwhile, the pressed residues were more enriched in V, Cr, Ni and Pb. Cd was also preferentially accumulated in the solid fractions, as it has been previously suggested to be a consequence of the addition of bentonites for wine clarification [20]. However, in the present work, the winemaking process did not include this step. Therefore, additional reasons, such as the association of this element to the protein related products, could be argued.

Chromium is retained in skins and seeds in pressed pomace, and its content does not change during clarification. Instead, the Cr concentration in wine uses to grow once bottled, because of the presence of chromium oxides in the glass pigments used [24]. However, this source of Cr could be discarded in the present work, as the wines analyzed were not previously bottled. The Pb concentration was around 10–30 times lower in musts than in the grape skin, which is in agreement with other results [25]. This element was accumulated in the pressed pomace, likely, because it mainly occurs as non-soluble complexes (pectic polysaccharide rhamnogalacturonan (II)) that are not degraded during the winemaking process, whereas free lead precipitates as PbS or as insoluble complexes with wine proteins [26,27]. This would explain the depletion in Pb concentration from must to wine.

Finally, it is worth mentioning that, by considering only the liquid fractions (bold characters in Table 1; Table 2), it was found that the elemental richest fraction corresponded to the must initially obtained by grape pressing. It was interesting to notice that, for elements such as B, Mg, Mn, Sr and Ba, all of them present at relatively high contents, the concentration did not modify or slightly decrease as the must evolved to wine. The concentrations for other elements, including P, Ca, V, Cr, As, Se, Hg, were around 50% of those found in the must, once the alcoholic fermentation was completed. Finally, elements such as Al, Fe, Cu, Zn, Ti, Ni, Co, Cd and Pb were drastically affected by the fermentation and, with some exceptions, their concentrations were from two to 12 times lower than those registered when analyzing the fresh must. An inverse relation between the Fe, Cu or Mn concentration and the flavonoids content has been assigned to the complex’s formation [28], which may further precipitate. Any deviation from this trend could be attributed to their incorporation to the must or wine from yeasts or components of the production line [29].

The obtained results were quite in agreement with the literature. Thus, Castiñeira-Gómez et al. [30] found that the boron content for white wine was from 10 to 40% below that in must. Similar qualitative trends for other elements (e.g., Cu and Ba) were also reported. Ca and Mg concentrations increased along the winemaking process. This was not in concordance with the results found in the present work, and could be accounted for by the fact that bentonites, that slightly increased the amount of these metals in the final wine [29], were not added. Therefore, possible discrepancies could be assigned to modifications in the vinification process. In our case, it was clearly confirmed that the most determining step from the point of view of elemental depletion in liquid fractions was the alcoholic fermentation.

The data in parenthesis presented in Table 1; Table 2 correspond to the maximum allowable levels, according to the OIV [7]. It may be observed that, for all the elements, except for Cu, the determined levels were below the maximum ones. Copper was likely present in the final wine, because it was employed as pesticide. In addition, it has been indicated that copper concentration in amateur winemaking (as that followed in the present work) may be higher than that for professional vinification [31]. Cu is an interesting element also from the point of view of fermentation efficiency, because it has been claimed that the yeast activity may be negatively affected for copper concentrations far above 30,000 μg kg^−1^ [32]. Fortunately, for the copper concentration indicated in Table 1 (around 15,700 μg kg^−1^), autochthonous yeast was expected to behave in a satisfactory way in terms of must fermentation. Expectedly, this yeast was able to accumulate such copper levels.

Regarding solid residues, the data reported in Table 1; Table 2 clearly demonstrated that metals tend to accumulate in grape marc. Composts prepared from this residue are beneficial for soil. However, the high content of metals should be taken into account, because it may be detrimental from an environmental point of view [33]. Thus, for instance, while arsenic concentration in musts and wine is on the order of 2–4 μg kg^−1^ (see Table 2 and reference [34]), its content raised up to 50 μg kg^−1^ in pressed pomace. However, it has been reported that, generally speaking, these kind of residues have contents in heavy metals on the order, or below, the concentrations found in urban wastes or manures [8]. 

### 2.5. Evolution of the Content of Rare Earth Elements

Rare earth elements (REEs) are highly relevant for wine discrimination purposes [35]. In the present work, the evolution of these elements during the winemaking process was also evaluated (Table 3). The most abundant REEs in solid fractions were Pr and Nd, followed by Sm, Eu and Gd. Other elements also present at appreciable concentrations were Dy and Hf. Meanwhile, liquid fractions (Table 3) contained much lower concentrations of these elements. In fact, some of them were not detected in fermented must or pressed must. Interestingly, Gd was not found in any of the liquid fractions. Comparatively speaking, it was observed that, virtually for all the studied cases, the concentration was the highest in the pressed pomace, whereas wine contained the lowest amount of REEs. This trend was found for all the elements except for Hf. In this case, it was observed that its content was significantly higher in the hat fraction than in the remaining ones.

REEs concentration variations can be explained on the basis of bentonite addition [36]. However, in the present work, the clarification process was based on gravitational settling and, hence, the presence of parts-per-trillion of these species could be mainly attributed to their migration from soil to the grapes by absorption through the plant roots.

### 2.6. Influence of the Yeast Addition

The winemaking process was also performed after addition of yeast (*S. cerevisiae*), in order to evaluate the influence of these microorganisms on the metal content. By comparison with the contents found in presence and in absence of added yeast, it was verified that, for K, Na, B, Mg, Al, P, Ca and Fe, the addition of microorganisms did not have any significant effect on the concentration.

However, for Cu interesting observations were made. Thus, the addition of yeast induced a growth in its concentration in the hat fraction (9300 ± 500 and 4000 ± 400 μg kg^−1^ in the presence and in the absence of added yeast, respectively). This could be partially assigned to the copper present in the added yeast (5600 ± 250 μg kg^−1^). While the copper content for fermented must and the must after pressing (fractions A and C, respectively) did not change upon the addition of yeast, a higher copper concentration in wine was found in absence than in presence of added yeast (101 ± 11 μg kg^−1^ with added yeast and 2100 ± 130 μg kg^−1^ without added yeast). Therefore, the wine obtained after the addition of *S. cerevisiae* contained a copper concentration below the maximum OIV acceptable limits [7], whereas the vinification with autochthonous yeasts led to values of this concentration above the allowed limits (see Table 2). This trend was accompanied by an increase in the Cu concentration in the lees (2700 ± 15 and 1350 ± 12 μg kg^−1^ in the presence and in the absence of added yeast, respectively). Additional reasons for the enrichment of Cu in the solid fractions were linked to the formation of copper sulfide complexes that were settled in the tank [37]. This was promoted by the transformation of elemental sulfur in hydrogen sulfide by the yeast [38]. Alternatively, the obtained data demonstrated the copper bioaccumulation in the added yeast [39]. 

An analogous situation was found for Zn (Figure S3a). In this case, the concentration of this metal in *S. cerevisiae* was actually high (around 200000 μg kg^−1^, see Figure S1c). Taking into account the mass of added yeast, (120 g yeast/100 L must), this represented a non-negligible amount of zinc. This element was partially distributed in the yeast removed after pressing the solid residues and that present in the decanted solids or lees (Figure S2a).

Regarding trace elements, it was discovered that Ti, Cr, Mn, Co, Cd, Hg and Pb were preferentially present in the solid residues when yeast was added, rather than in its absence. Figure S2b shows the data obtained for cadmium. This element was present in yeast as impurities at a concentration of 14 μg kg^−1^. This could be considered to be the main reason for its buildup in solid fractions such as pressed and decanted residues. For other elements that were not present in yeast, such as mercury, it was found that its content in the hat did not depend on the addition of yeast. Nevertheless, the bioaccumulation capability of the yeast was evidenced, because the mercury concentration was higher in the pressed pomace and lees when yeast was added (16 ± 1 and 1.9 ± 0.2 μg kg^−1^, respectively) than without its use (11 ± 1 and 0.42 ± 0.03 μg kg^−1^, respectively). It was finally observed that V, Ni, As, Se, were present at similar concentrations regardless of the yeast addition.

### 2.7. Influence of the Vineyard

The grape composition had an obvious direct influence on the metallic load of the different fractions. To illustrate this fact, potassium was taken as representative element. Figure S3 shows the concentration of this element for the different fractions under study. It was observed that K was more abundant in the grapes originated from one of the two localizations evaluated (Alfafara) than for the alternative one (Alcocer). This promoted higher K concentrations in the remaining fractions. The same could be stated for all the elements measured. Therefore, elemental grape analysis is a good indication of the quality of the obtained wines and residues.

## 3. Discussion

### 3.1. Behavior of the Elements as a Function of the Fraction

**Figure 2:**
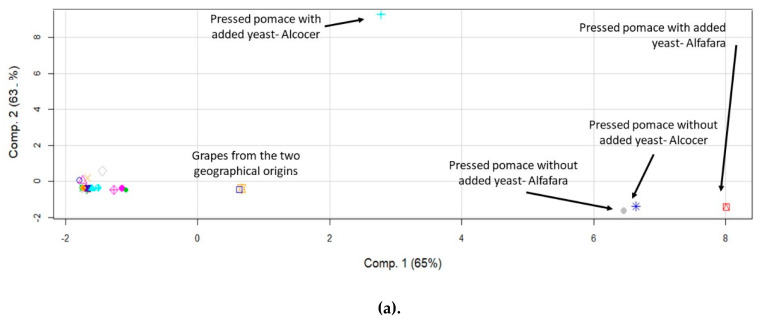

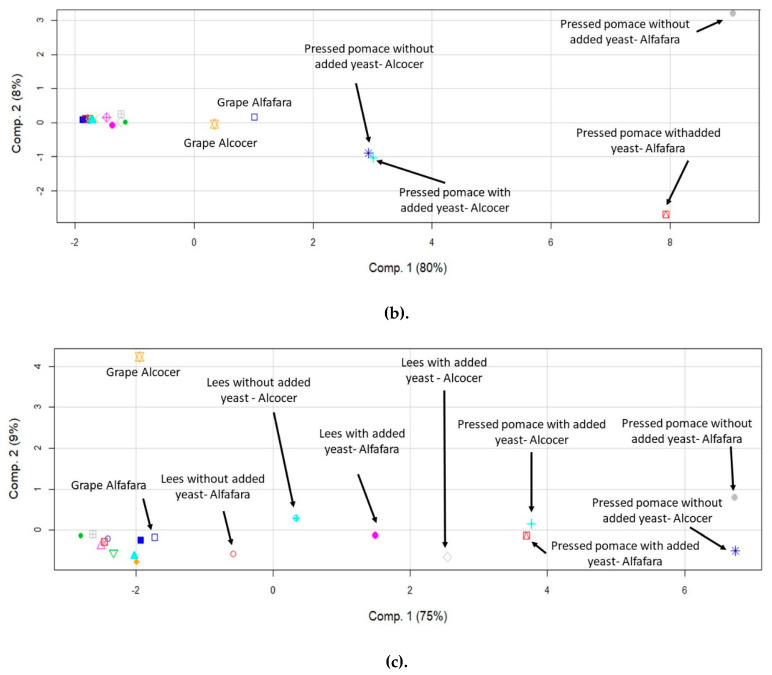
Score plots of the principal component analysis corresponding to the data expressed as concentration of elements for fractions of the winemaking process originated from two different geographical locations with and without yeast addition. (**a**) Major elements; (**b**) trace elements; (**c**) rare earth elements.

In order to study possible differences in the behavior of the elements in terms of their accumulation in the evaluated winemaking fractions, principal component analysis (PCA) was applied. Initially, the objects were the fractions while the variables were the determined elements. Previous reports have demonstrated the capability of this data treatment for discriminating wine samples according to their provenance [40]. PCA has also proven to be useful in order to trace the origin of the wines by analyzing berries, leaves and wines [41]. Figure 2 shows the score plots obtained for major (a), trace (b) and rare earth elements (c). The points on the graph correspond to the obtained results for the fractions, with and without yeast addition, and the two geographical origins.

By examining the data for major elements (Figure 2a), it may be observed that some samples separated, according to a component explaining 65% of the total variance of the elemental profile as a function of the fractions under study. Interestingly, pressed pomace and grapes separated from the rest, thus indicating that they had a differentiated elemental concentration profile. The same observation was made by evaluating the results for trace elements in which 80% of the total variance was explained by component 1 (Figure 2b). In both cases, i.e., major and trace elements, component 1 was able to discriminate the geographical origin of the pressed pomace. The situation found for rare earth elements, however, was significantly different, especially for lees. It was figured out that the concentration profiles for these elements were different, as compared to those for liquid fractions and hat. In this case, component 1 explained 75% of the data variability. Again, pressed pomace differentiated from the rest of the fractions, and it was possible to discriminate between the two origins. Rare earth elements, in turn, helped to discriminate between lees and pomace from the remaining fractions.

From the PCA analysis, it was concluded that the elemental concentration profiles for solid and liquid winemaking fractions were different. With the aim of determining what elements played the most critical role, a new PCA procedure was applied to the data in which the elements were considered as objects and the fractions were the variables. The main conclusions of this second study were that three major elements (Mg, P and K), four trace elements (Pb, Ni, Ba and Rb) and seven rare earth elements (Er, Pr, Gd, Sm, Eu, Hf and Nd) separated from the rest of analytes, according to a component that respectively explained the 55%, 92%, 48% (Alcocer) and 98%, 84% and 60% (Alfafara) of the total variance of the data.

### 3.2. Studies about the Origin of the Metals

In order to elucidate the origin of metals in the final products as well as to get better control on the winemaking process, a mass balance was established considering the mass of the different elements in each one of the fractions on a wet basis. Therefore, the moisture was determined in the case of solid residues. In addition, according to experimental measurements, 70% of the initial grape mass corresponded to water, 27% to solid residues and 3% was the fraction of decanted mass (lees). These data were quite in agreement with others previously published [20]. Table 4 shows the results found for potassium taken as an example. It was concluded that the total mass of this element considering the different fractions was similar to the sum of the mass of potassium supplied by grapes and yeast. There was only an around 3% deviation between these two magnitudes. It was, hence, possible to conclude that there were neither losses nor contaminations of this element during the winemaking process.

(3) $$*Deviation=\frac{\left(Sum-mass_{supplied\;by\;grapes\;+yeasts}\right)}{mass_{supplied\;by\;grapes+yeasts}}\times 100$$

**Figure 3:**
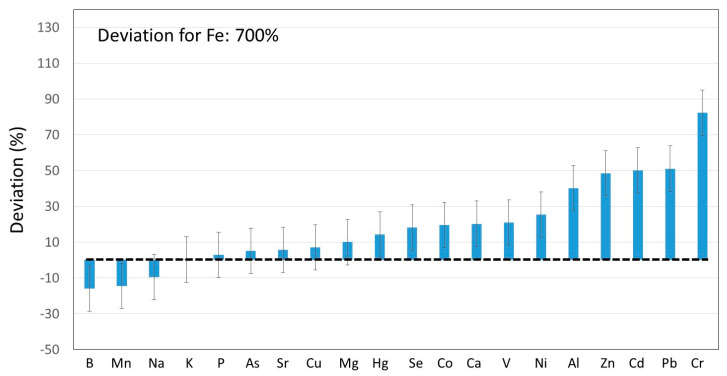
Deviation percentages between the elemental mass contained in the different fractions of the winemaking process, with respect to the mass contained in the grapes and yeast.

The same consideration was thus extrapolated to other elements. The obtained results are summarized in Figure 3, for a total of 21 elements. First, it should be noted that the precision associated with the so-called deviation, calculated from error propagation, was 20% (see error bars in Figure 2). Therefore, for 14 out of the twenty-one elements tested (i.e., B, Mn, Na, K, P, As, Sr, Cu, Mg, Hg, Se, Co, Ca and V), the mass found in the different fractions corresponded to the mass originally present in the grapes + yeast. Nevertheless, for Ni, Al, Zn, Cd, Pb, Cr and especially for Fe, the deviation was significantly higher than 0. This fact suggested that these elements were incorporated to the products during the grape processing.

Previous reports have anticipated this kind of trends. Thus, elements such as chromium, cadmium and lead became more concentrated during the wine maturation [42]. The release of these elements from stainless steel and brass cellar equipment was claimed to be in the origin of this observation. In other studies, it has been indicated that traditional vinification processes may lead to the incorporation of elements such as Cd, Cr, Cu, Fe, Ni, Pb, V and Zn [38]. These results were also in agreement with those reported in Figure 3 for this list of elements except for copper.

The huge increase in Fe content, as compared to the expected one according to its concentration in grape and yeast (deviation: 700%), could be attributed to its migration from the stainless steel used in the production plant (conductions, tanks). The same could be applied to Zn, since it can be present at low levels in this kind of material. Aluminum, in turn, may be present in the final wine if it is in contact with surfaces containing the employed allow [42]. For chromium, it has been previously observed that its concentration increased with the wine aging and storage. This was also attributed to the fact that wine was in contact with stainless steel containers. 

A similar treatment was done for REEs and it was observed that the deviations as defined in Table 3 footnote were lower than 30% for Sm, Eu, Gd, Dy and Yb (i.e., 5.7%, 9.9%, 15%, 3.9% and 24%, respectively). This result was considered to be quite satisfactory taking into account the relative error of the concentration for this kind of elements. However, for Pr and Nd, this parameter took values of 64% and 61%, respectively. Ho, Er, Tm, Lu and Hf were not detected when the grapes were analyzed. Although the concentrations of these elements were actually low, it was observed that mass balance was correct for almost all the REEs detected in grapes.

All these results collectively confirmed the hypothesis initially emitted and it was verified that the mass balance contributed to unequivocally discerning whether the winemaking process was itself a source of metals and REEs.

## 4. Materials and Methods

### 4.1. Winemaking Process

**Figure 4:**
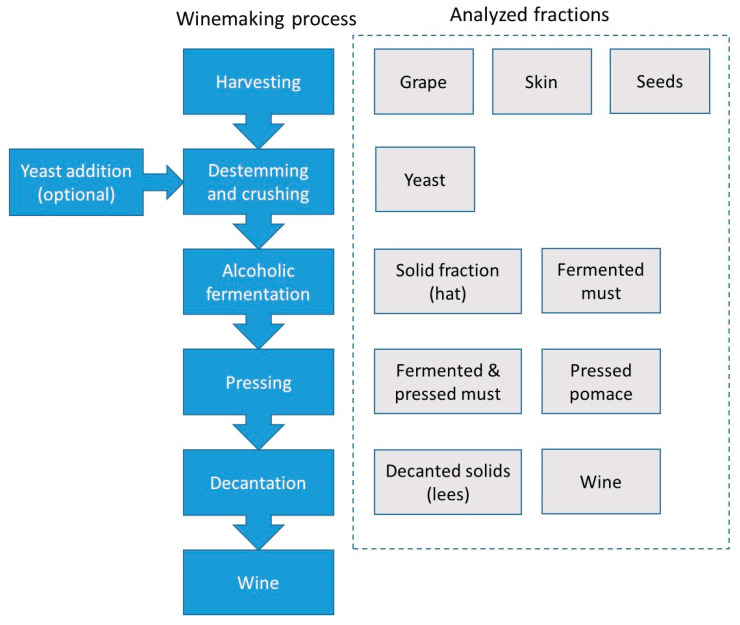
Scheme of the winemaking process (blue boxes) and analyzed fractions (grey boxes).

The amateur winemaking method followed in the present work is schematized in Figure 4. The cultivars used to produce the wine consisted in a mixture of *Vinitis vinifera Tempranillo* and *Vinitis vinifera Grenache* in a 4:1 weight proportion. After the harvesting, the grapes were de-stemmed and crushed with an automatic device. Then, the fermentation started in 200 L stainless steel containers around 12 h after the storage of the grapes-must mixture. This step was followed in two different ways: without and with the yeast addition. In the second situation, 120 g of commercial cultured *S. Cerevisiae* were added per 100 L total volume. Alcoholic fermentation was completed after 8 days, and then the mixture was mechanically pressed. During this period of time, the solid fraction containing skin and seeds accumulated at the uppermost part of the container, thus constituting the so-called hat. In order to improve the migration of anthocyanins, this solid fraction was sunken twice a day. After the alcoholic fermentation was completed, the so-called hat fraction was taken for further analysis. Once the pressed pomace was separated from the must, the liquid turbid fraction was inserted again in the steel container, and left for two months. During this period of time, air was prevented from entering into contact with the fermented and pressed must. The malolactic fermentation took place and this fact was verified by means of a thin layer chromatography assay. During this period of time, suspended solid matter containing dead yeasts, proteins and tannins, was naturally decanted and accumulated at the bottom of the container. The liquid clear fraction was thus bottled. Wine was eventually exposed to air what decreased tannin content and increased the wine fruitiness. In order to follow the evolution of the metal content throughout the winemaking process, the fractions highlighted in Figure 4 were sampled and analyzed.

The grapes originated from two different but close vineyards located in the north of Alicante, Spain. Location A corresponded to Alcocer (Latitude: 38.7950; longitude: −0.402440), whereas location B was situated in Alfafara (Latitude: 38.7727; longitude: −0.573399). 

### 4.2. Reagents, Samples and Sample Preparation

Nitric and sulfuric acids (Suprapur^®^ Merk, Darmstadt, Germany) were used. In addition, multielemental standards were prepared from a 1000 mg L^−1^ stock solution (Merk IV). 

All the samples were analyzed after a previous drying and microwave acid digestion step in sealed PTFE reactors using a Milestone oven (Sart D, Sorisole, Italy). A total of 2 g of raw solid fractions (yeast, grape, skin, seed, hat, pressed pomace and lees) were weighed and inserted into a dryer at 100 °C overnight. The solid residue was again weighed into the PTFE reactors. Regarding the liquid samples (fermented must, fermented and pressed must and wine), they were directly put into the reactors without a previous drying step. A total of 7 mL of nitric acid and 1 mL of sulfuric acid were poured into the PTFE reactors. The reactors were sealed and adapted to the oven carousel. Sample digestion was performed at a 200 °C temperature and 45 bar maximum pressure for 15 min, followed by a 110 °C and 75 bar treatment for 15 additional minutes. To achieve these conditions, the microwave power was set at 1500 W. Once the samples were digested, the solutions were allowed to cool down for 45 min, and they were subsequently diluted with ultrapure water (Millipore, El Paso, TX, USA) up to 25 g. All these experiments were done by quadruplicate. 

### 4.3. Analysis of the Digests

Multielemental analyses were performed by means of an inductively coupled plasma mass spectrometer (ICP-MS, Agilent, 7700x, Santa Clara, CA, USA). Some of the experiments were performed with an inductively coupled plasma optical emission spectrometer (ICP-OES, Agilent, 5100, Santa Clara, CA, USA). Table 5 summarizes the operating conditions set for both instruments.

Principal component analysis (PCA) was carried out using the open-source software R [43] version 3.5.3 (Vienna, Austria), with the additional packages Rcdmr [44] and CAT [45].

## 5. Conclusions

From the obtained results, several conclusions were drawn. As expected, the most abundant elements in the winemaking fractions are K, Na, Mg, P and Ca. With regards to transition metals, Cu, Ti and Ni have been the most concentrated elements, likely because of their use in phytosanitary treatments, must pollution or assimilation through the plant roots. Finally, Nd was the rare earth element present at the highest concentration in virtually all the evaluated fractions. The selected detection technique (i.e., ICP-MS) showed several advantages over other quantification methods, such as energy dispersion X-ray spectroscopy, which has the capability of achieving extremely low detection limits although the sample had to be dissolved, prior to its analysis.

The present work demonstrates that most of the elemental content was found in the solid fractions of the winemaking process. Among them, pressed pomace contained the highest load of major and trace elements as well as rare earth elements. This was in direct link with the elemental repartition along the grape parts. Thus, it was verified that the skin was the richest bay fraction in terms of metal concentration. There were some confirmed exceptions to this rule (e.g., Ni or Cu) that highlighted the complex chemical process occurring during the wine production. The results also demonstrated the active role of yeast in scavenging some elements (e.g., Cu, Hg) through bioaccumulation.

A principal component analysis revealed that the elemental profiles of grapes, pressed pomace, and lees differed from those for musts, wine and hat. This data treatment method also made it possible to identify the geographical source of a given winemaking fraction. The elements most responsible for this discrimination were Mg, P, K (as major) Pb, Ni, Ba, Rb (as trace) and Er, Pr, Gd, Sm, Eu, Hf and Nd (as rare earth elements). 

The results obtained when applying a procedure based on the determination of the mass balance for each particular element confirmed the hypothesis that this procedure was extremely useful to control the quality and safety of wine production. Elements such as Ni, Al, Zn, Cd, Pb and Cr could migrate from the components of a wine production plant to the must. 

Further research is needed to obtain additional information about the chemical form in which the different elements are present in each one of the fractions of the winemaking process. Additional cultivars, geographical areas and post-production processes are also of interest, in order to optimize the best practices and varieties from the point of view of quality and environmental safety. Other fermentation processes (e.g., beer and cider production) could benefit from this kind of study.

## Figures and Tables

**Table 1:** Concentrations (μg kg^−1^) found for abundant elements in the different fractions of the winemaking process without added yeast *.

	B	Na	Mg	Al	P	Ca	Fe	Mn	Sr	Ba	Cu	Zn
**O**	**20400 ± 15**	**114284 ± 15800**	**509700 ± 10200**	**8789 ± 807**	**1189100 ± 198000**	**282814 ± 36300**	**12024 ± 786**	**4453 ± 205**	**4100 ± 200**	**652 ± 120**	**3930 ± 110**	**4110 ± 800**
**A**	**4141** **± 163**	**10059** **± 321**	**112665** **± 1700**	**6366** **± 571**	**276610** **± 13000**	**28265** **± 3979**	**6591** **± 662**	**613** **± 19**	**493** **± 37**	**45 ± 2**	**15747** **± 778**	**6706** **± 132**
**B**	**3876** **± 213**	**5235** **± 321**	**114086** **± 860**	**740** **±** **368**	**127081** **±** **1204**	**18018 ± 1974**	**735 ± 153**	**560 ± 5**	**419 ± 6**	**46 ± 5**	**220 ± 87**	**730 ± 142**
C	26365 ± 2980	42434 ± 5410	684204 ± 86370	17025 ± 1981	1013301 ± 1670	354918 ± 6033	24295 ± 1289	4594 ± 592	13219 ± 1802	3162 ± 53	3972 ± 401	5142 ± 370
**D**	**3837** **± 100**	**4602** **± 598**	**115544** **±** **1282**	**550** **±** **279**	**144600** **±** **1702**	**16778 ± 1503**	**511 ± 3**	**531 ± 7**	**416 ± 7**	**46 ± 4**	**163 ± 9**	**1012 ± 171**
E	32080 ± 2935	16227 ± 2820	1039619 ± 110768	83940 ± 2375	3268118 ± 295792	1023100 ± 139161	132907 ± 4443	11555 ± 1976	14421 ± 7	3162 ± 53	14768 ± 1009	12467 ± 682
**F**	**3776** **± 348 (80000)**	**5494** **± 560**	**116005 ± 4088**	**1086** **±** **139 (10000)**	**158364** **±** **3721**	**19217 ± 1138**	**2086 ± 404 (20000)**	**583 ± 21**	**460 ± 12**	**58 ± 4**	**2074 ± 130 (1000)**	**1824 ± 445 (5000)**
G	4216 ± 195	19265 ± 378	108524 ± 995	4017 ± 1491	222788 ± 6742	23322 ± 7411	3222 ± 876	639 ± 13	437 ± 27	74 ± 16	1350 ± 167	1092 ± 143

* O: Grape; A: Must; B: Fermented must; C: Hat; D: Fermented & pressed must; E: Pressed pomace; F: Wine; G: Decanted solids or lees. Bold characters correspond to liquid winemaking fractions. The figures in parenthesis show the maximum acceptable limits according to the International Organization of Vine and Wine (OIV). Confidence intervals were calculated according to: $\pm \;\frac{t\;s}{\sqrt{n}}$ (*n* = 4; t for a 99% confidence level: 3.74).

**Table 2:** Concentrations (μg kg^−1^) found for trace elements in the different fractions of the winemaking process without added yeast *.

Fraction	Ti	Ni	V	Cr	Co	As	Se	Cd	Hg	Pb
O	372 ± 97	230 ± 50	63 ± 18	25 ± 1	12 ± 2	8.2 ± 0.5	20 ± 3	6.2 ± 1.5	4.8 ± 0.2	24 ± 9
**A**	**121 ± 28**	**1427 ± 27**	**29 ± 3**	**32 ± 1**	**18.8 ± 0.6**	**4.3 ± 0.2**	**7.9 ± 0.2**	**2.0 ± 0.2**	**0.7 ± 0.2**	**190 ± 9**
**B**	**25 ± 5**	**93 ± 8**	**18 ± 3**	**19 ± 9**	**4.36 ± 0.03**	**0.9 ± 0.1**	**5 ± 1**	**0.58 ± 0.04**	**0.5 ± 0.1**	**12 ± 5**
C	941 ± 45	259 ± 25	94 ± 11	59 ± 10	12.9 ± 0.8	24 ± 3	426 ± 24	1.46 ± 0.02	30 ± 3	36 ± 11
**D**	**18 ± 5**	**91 ± 2**	**21 ± 4**	**21 ± 2**	**4.78 ± 0.11**	**1.9 ± 1.1**	**4 ± 1**	**0.57 ± 0.02**	**0.2 ± 0.1**	**7 ± 1**
E	3368 ± 135	281 ± 81	204 ± 27	299 ± 79	37 ± 5	50 ± 3	63 ± 14	4.6 ± 0.3	12 ± 2.0	129 ± 29
**F**	**54 ± 2**	**94 ± 4(100)**	**29 ± 3**	**14 ± 3(100)**	**3.7 ± 0.2**	**2.4 ± 0.2(200)**	**5 ± 1**	**2.0 ± 0.2(10)**	**0.4 ± 0.2**	**< LOD(100)**
G	431 ± 68	61 ± 3	18 ± 2	13 ± 3	3.9 ± 0.2	3.2 ± 0.6	9 ± 1	0.77 ± 0.03	0.4 ± 0.1	13 ± 3

* O: Grape; A: Must; B: Fermented must; C: Hat; D: Fermented & pressed must; E: Pressed pomace; F: Wine; G: Decanted solids or lees. Bold characters correspond to liquid winemaking fractions. The figures in parenthesis show the maximum acceptable limits according to the International Organization of Vine and Wine (OIV). The level for lead has been established in the 2019 summary of resolutions adopted in 2019 by the 17th General Assembly of the OIV—Geneva (Switzerland). Confidence intervals were calculated according to: $\pm \;\frac{t\;s}{\sqrt{n}}$ (*n* = 4; t for a 99% confidence level: 3.74).

**Table 3:** Concentrations (μg g^−1^) found for trace elements in the different fractions of the winemaking process without added yeast *.

Fraction	Pr	Nd	Sm	Eu	Gd	Tb	Dy	Ho	Er	Tm	Yb	Lu	Hf
O	8.8 ± 1.7	25 ± 2	20 ± 3	16 ± 3	10.0 ± 0.2	1.3 ± 0.2	14 ± 2				7.9 ± 0.5		
**A**	**160 ± 20**	**292 ± 18**		**141 ± 26**		**16 ± 2**	**96 ± 9**	**21 ± 2**	**33 ± 2**	**13 ± 2**	**84 ± 6**		**228 ± 40**
**B**	**69 ± 13**			**104 ± 15**		**19 ± 2**	**164 ± 5**	**36 ± 7**		**31 ± 5**			**600 ± 10**
C	1415 ± 62	4276 ± 200	960 ± 80	864 ± 60	1512 ± 50	131 ± 26	410 ± 65	39 ± 4	325 ± 52	50 ± 6	385 ± 70	145 ± 22	2836 ± 130
**D**	**67 ± 8**	**244 ± 41**	**351 ± 17**	**135 ± 15**				**35 ± 6**	**56 ± 5**				**432 ± 80**
E	4580 ± 212	10060 ± 210	1900 ± 420	952 ± 50	2518 ± 260	250 ± 15	1693 ± 320	57 ± 5	488 ± 75	87 ± 17	532 ± 102	123 ± 22	1120 ± 205
**F**	**46 ± 7**	**300 ± 45**	**161 ± 30**	**166 ± 22**					**102 ± 21**		**65 ± 13**		**375 ± 61**
G	705 ± 133	2140 ± 120	760 ± 25	332 ± 52	840 ± 57	55 ± 2	303 ± 14	57 ± 2	220 ± 42	44 ± 1	89 ± 2	77 ± 13	292 ± 34

* O: Grape; A: Must; B: Fermented must; C: Hat; D: Fermented & pressed must; E: Pressed pomace; F: Wine; G: Decanted solids or lees. Bold characters correspond to liquid winemaking fractions. The figures in parenthesis show the maximum acceptable limits according to the International Organization of Vine and Wine (OIV). Confidence intervals were calculated according to: $\pm \;\frac{t\;s}{\sqrt{n}}$ (*n* = 4; t for a 99% confidence level: 3.74).

**Table 4:** Mass balance for potassium ^#.^

Fraction	Yield (%)	Concentration (mg/kg) ^#^	% Water	Total Mass of Potassium (mg)
Pressed pomace	27	33800	80	1825
Lees	3	3100	85	14
Wine	70	1477	100	1034
Sum				2873
grapes		11100	75	2775
yeasts		13100		20
Mass supplied by grapes + yeasts				2795
Deviation (%) *				3.4

^#^ The calculation basis corresponded to 1 kg.

**Table 5:** Inductively coupled plasma optical emission spectrometer (ICP-OES) and inductively coupled plasma mass spectrometer (ICP-MS) instrumental conditions.

**ICP-OES**	
**Variable**	**Values and Unities**
Nebulizer liquid flow rate	1.0 mL min^−1^
Nebulizer gas flow rate	0.7 L min^−1^
Outer plasma gas flow rate	15 L min^−1^
Intermediate plasma gas flow rate	1.5 L min^−1^
RF power	1.4 kW
**ICP-MS**	
**Variable**	**Values and Unities**
Nebulizer liquid flow rate	0.4 mL min^−1^
Nebulizer gas flow rate	0.7 L min^−1^
Outer plasma gas flow rate	15 L min^−1^
Intermediate plasma gas flow rate	1.0 L min^−1^
RF power	1.6 kW
He (Collision cell) gas flow rate	4.3 mL min^−1^

## Supplementary Materials

The following are available online; Figure S1: Elemental concentration referred to dry mass for (a) and (b) grape, grape seeds and skin and, (c) yeast. For figs (a) and (b) Black bars: grape; white bars: seeds; grey bars: skin. The bars correspond to the mean of four replicates; Figure S2: Comparison of the evolution of zinc (a) and cadmium (b) concentration with and without added yeast. White bars: process with addition of *S. Cerevisiae*; grey bars: process carried out with only autochthonous yeast; Figure S3: Potassium concentration for the vinification fractions corresponding to two different grape origins. Black bars: Alfafara; grey bars: Alcocer; full bars: without added yeast; dashed bars: with added S. Cerevisiae; Table S1: ICP-MS limits of detection (LOD), and method limits of quantification (mLOQ) for the elements determined.
