# Supplementary material for: Evolution of the Multielemental Content along the Red Wine Production Process from Tempranillo and Grenache Grape Varieties
Source: Molecules. 2020 Jun 27;25(13):2961. doi: 10.3390/molecules25132961 (PMC7411754; doi:10.3390/molecules25132961)
Supplement: Supplementary file 1 [file molecules-25-02961-s001.pdf]

Article

Evolution of the Multielemental Content along the Red Wine Production Process from *Tempranillo* and *Grenache* Grape Varieties

Alexandra Bica, Raquel Sánchez and José-Luis Todolí *

Department of Analytical Chemistry, Nutrition and Food Science, University of Alicante, P.O. Box 99, 03080 Alicante, Spain; alexandra.bica@gmail.com(A.B.); r.sanchez@ua.es(R.S.)

* Correspondence: jose.todoli@ua.es

Academic Editor: Jose Miguel Hernandez-Hierro
Received: 25 May 2020; Accepted: 24 June 2020; Published: 27 June 2020

Supplementary Materials

Table S1. ICP-MS limits of detection, LOD, and method limits of quantification, mLOQ, for the elements determined.

	LOD (μg kg^{-1})	Method LOQ (μg kg^{-1})		LOD (μg kg^{-1})	mLOQ (μg kg^{-1})
	Major elements			Rare earth elements	
B	2	87	Pr	0.002	0.08
Na	2	87	Nd	0.007	0.3
Mg	3	125	Sm	0.002	0.08
Al	0.3	11	Eu	0.001	0.04
P	30	1250	Gd	0.002	0.08
Ca	2	87	Tb	0.002	0.08
K	31	1300	Dy	0.001	0.04
Fe	0.02	0.9	Ho	0.001	0.04
Mn	0.3	12	Er	0.001	0.04
Sr	0.03	1.4	Tm	0.001	0.04
Ba	0.02	1.0	Yb	0.001	0.04
Cu	0.12	5.0	Lu	0.001	0.04
Zn	0.2	7.5	Hf	0.003	0.12
Rb	0.01	0.4			
Mo	0.01	0.6			
		Trace elements			
Ti	0.05	2.5	As	0.01	0.4
Co	0.003	0.1	Se	0.2	7.5
Ni	0.3	11	Cd	0.01	0.3
V	0.003	0.1	Hg	0.012	0.5
Cr	0.04	1.5	Pb	0.12	1.9

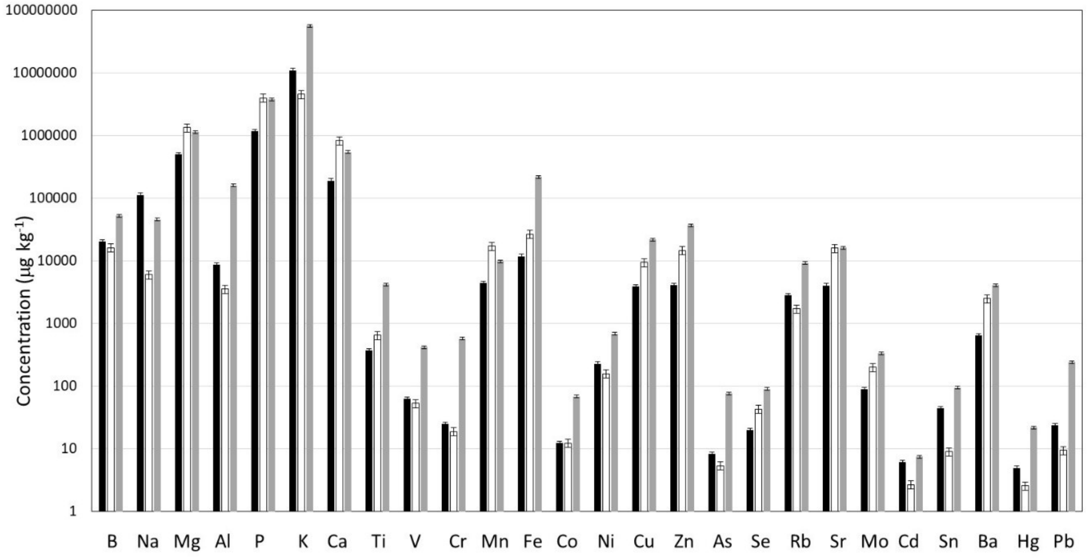

100000000
10000000
1000000
100000
10000
1000
100
10
1
Concentration (µg kg-1)
B
Na
Mg
Al
P
K
Ca
Ti
V
Cr
Mn
Fe
Co
Ni
Cu
Zn
As
Se
Rb
Sr
Mo
Cd
Sn
Ba
Hg
Pb

(a).

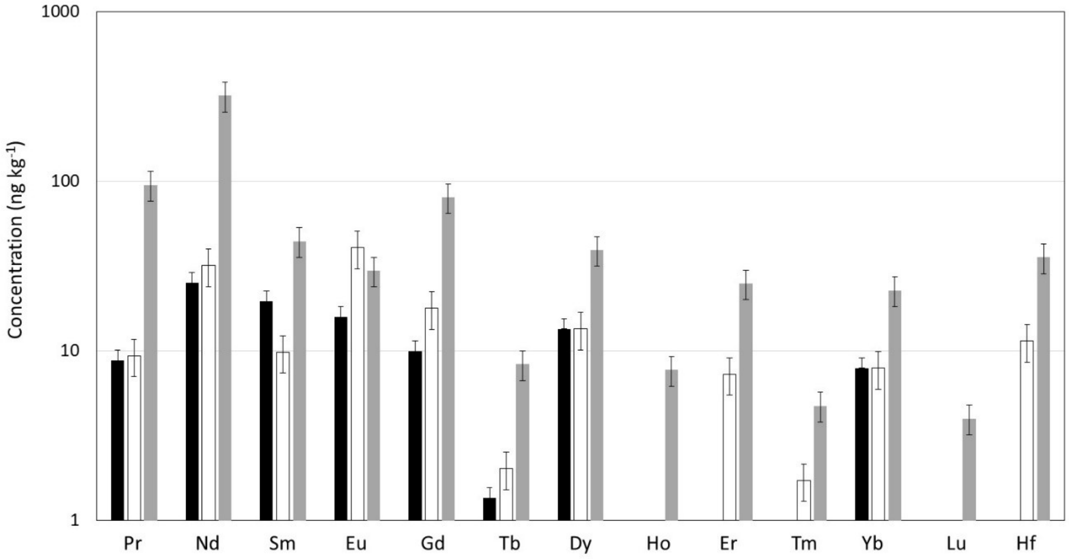

1000
100
10
1
Concentration (ng kg-1)
Pr
Nd
Sm
Eu
Gd
Tb
Dy
Ho
Er
Tm
Yb
Lu
Hf

(b).

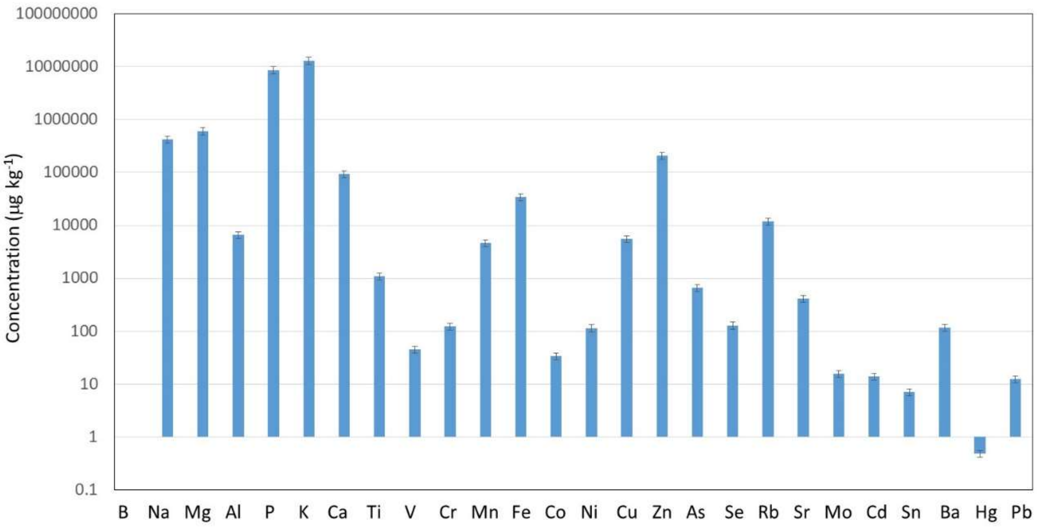

(c).

Figure S1. Elemental concentration referred to dry mass for (a) and (b) grape, grape seeds and skin and, (c) yeast. For figs (a) and (b) Black bars: grape; white bars: seeds; grey bars: skin. The bars correspond to the mean of four replicates..

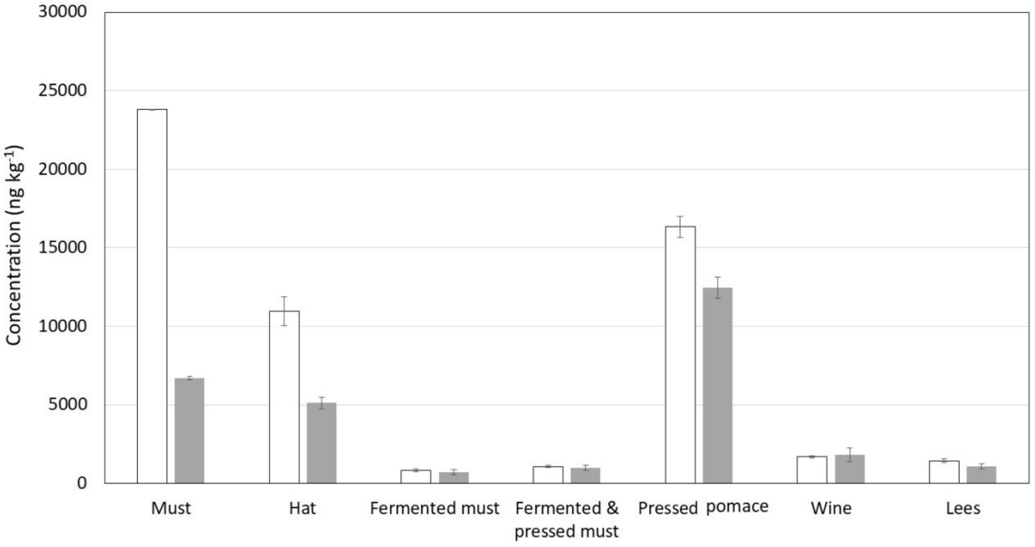

(a).

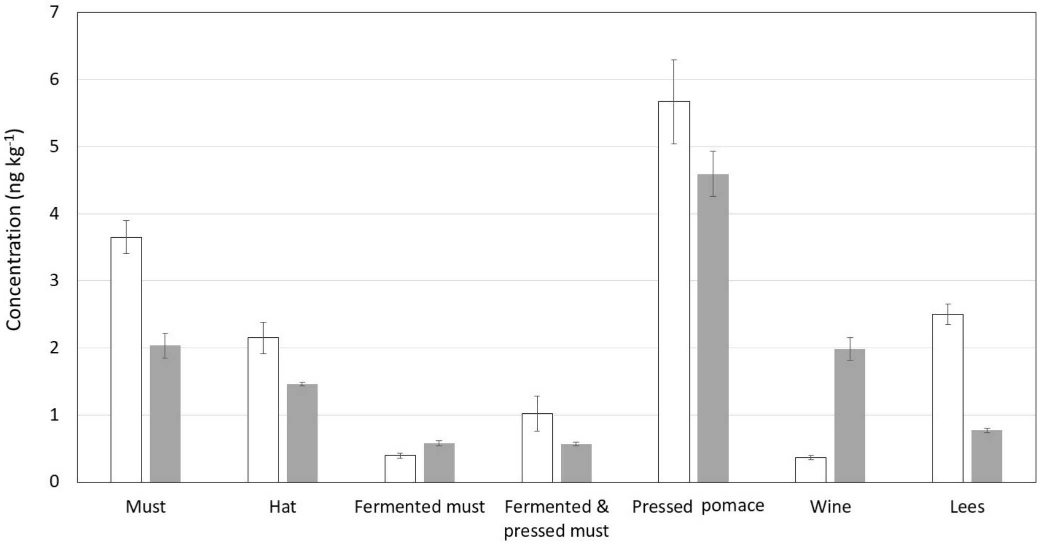

(b).

Figure S2. Comparison of the evolution of zinc (a) and cadmium (b) concentration with and without added yeast. White bars: process with addition of *S. Cerevisiae*; grey bars: process carried out with only autochthonous yeast.

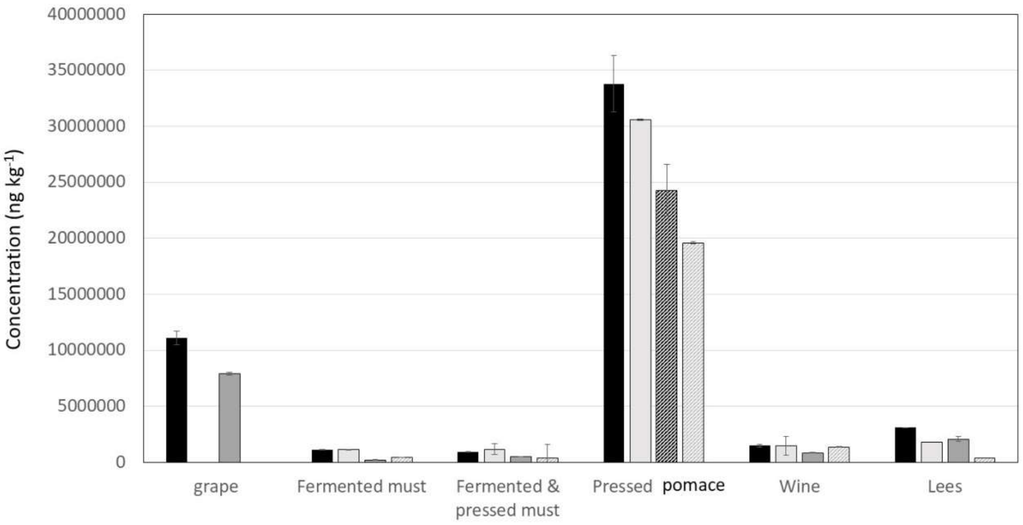

Figure S3. Potassium concentration for the vinification fractions corresponding to two different grape origins. Black bars: Alfafara; grey bars: Alcocer; full bars: without added yeast; dashed bars: with added *S. Cerevisiae*.
